# Reliability and criterion validity of abductor hallucis muscle volume measurements using ultrasound and MRI by assessors with limited image analysis experience

**DOI:** 10.1371/journal.pone.0360725

**Published:** 2026-10-21

**Authors:** Jacob B. Willes, Emma Robinson, Joshua K. Sponbeck, Derek A. Swanson, Dallin C. Swanson, Austin G. Goodrich, Steven P. Allen, A. Wayne Johnson

**Affiliations:** 1 Department of Exercise Sciences, Brigham Young University, Provo, Utah, United States of America; 2 Department of Athletic Training and Occupational Therapy, Weber State University, Ogden, Utah, United States of America; 3 Arizona College of Podiatric Medicine, Midwestern University, Glendale, Arizona, United States of America; 4 John Sealy School of Medicine, The University of Texas Medical Branch at Galveston, Galveston, Texas, United States of America; 5 Department of Electrical and Computer Engineering, Brigham Young University, Provo, Utah, United States of America; University of Mississippi, UNITED STATES OF AMERICA

## Abstract

**Purpose:**

The abductor hallucis (ABDH) contributes to foot function and mobility, which can influence quality of life, making accurate assessment of muscle volume important. While magnetic resonance imaging (MRI) is often considered to be the reference standard of medical imaging, ultrasound (US) provides a cost-effective, rapid and dynamic way of assessing muscle size and function. This study aimed to evaluate the criterion validity and intrarater reliability of US-derived ABDH muscle volume measurements compared with MRI when image analysis was performed by assessors with limited experience.

**Methods:**

Us And MRI were employed to measure the right and left ABDH muscle volume involving 22 participants (females = 12; males = 10). Following exclusions, 37 feet were available for analysis, with 36 complete MRI-US observations. Muscle volume of the ABDH was calculated across the muscle belly every 0.5 cm both manually by utilizing a truncated cone (TC) formula and with a semi-automated tool in the OsiriX (OX) software. Intrarater reliability was assessed using intraclass correlation coefficients (ICC), standard error of measurement (SEm), and minimal detectable change (MDC). Criterion validity was evaluated using Pearson correlations, Bland-Altman analyses, and two one-sided tests (TOST) with a determined ±10% equivalence margin.

**Results:**

Side-specific intrarater reliability was high across methods (ICC(3,1) = 0.932–0.997), with SEm values of 0.251–0.709 cm³ and relative MDC values of 4.48%−12.95%. Very strong associations were observed among methods (r = 0.906–0.995). Bland-Altman limits-of-agreement half-widths were 6.52%−9.84% for MRI-MRI and 17.86%−27.35% for MRI-US comparisons. All three method comparisons met the ± 10% equivalence criterion.

**Conclusion:**

US-derived ABDH volume measurements demonstrated high intrarater reliability and statistical equivalence to MRI within the ± 10% margin. Although MRI-US comparisons showed greater individual-level variability than MRI-MRI comparisons, US may provide a practical alternative for assessing ABDH muscle volume when MRI is impractical.

## Introduction

Foot muscles are vital in contributing to various functions of the human body [1] such as walking and are directly correlated with quality of life [2]. These muscles provide support and stability for the body during stance [3] and bipedal locomotion [4]. This is achieved in part by maintaining the arches of the foot [5], facilitating movement [6], and providing shock absorption [7]. The abductor hallucis (ABDH) is one of the largest intrinsic muscles of the foot and a key muscle in maintaining and controlling the medial longitudinal arch [8]. Weakness and atrophy of intrinsic and extrinsic muscles have been linked to the development of lower extremity conditions such as pronounced pain [2,9], plantar fasciopathy [10], acquired pes planus [11], diabetic neuropathy [12] and additionally, can lead to detrimental stresses in other parts of the body [13]. Thus, being able to accurately assess these muscles, particularly the ABDH, is important to improve performance of the foot, provide targeted rehabilitation exercises, and support the overall health of the person [1].

Measuring intrinsic foot muscles has been shown to be a reliable method of determining muscle function and condition [14]. Ultrasound (US) and magnetic resonance imaging (MRI) are both non-invasive methods that are commonly employed to accurately measure and assess intrinsic foot muscle size [15]. The use of MRI is widely accepted in medical practice as the reference standard for the imaging of body structures [16]. MRI provides high spatial resolution and functional imaging, enabling detailed visualization and contrasting of small structures and other fine anatomical details. Additionally, multiplanar imaging allows for a comprehensive examination from different angles, providing a more complete understanding of complex structures. However, there are limitations to this modality compared to US. MRI is much more expensive, time consuming, less readily available than US [17] and does not allow for muscle contraction during imaging. US has the unique ability to capture images in real time, such as when a muscle is being contracted versus relaxed, which is particularly valuable when assessing muscle function and measuring muscle cross-sectional area (CSA) [18].

When choosing an appropriate modality for measuring and assessing foot muscles, it is important to recognize the challenges associated with identifying muscle size, strength and function, such as the interplay between a person’s body composition and muscle architecture [19]. Although muscle measurement has traditionally been done by muscle thickness and CSA, these measurements are limited to one or two dimensions [20]. Volume and shape are strongly correlated with function [21] and can be measured accurately through an auto volume segmentation software or truncated cone formula [22]. In recent research, calculating muscle volume has been reliable for the evaluation of size-strength relationships and the existence of age-related difference in muscle strength per size, showing a significant correlation of torque with muscle volume in both young and elderly individuals [23]. Thus, muscle volume is a reliable source for evaluating size-strength relationship and the existence of age-related difference in muscle strength [23].

Another critical factor in assessing intrinsic foot muscles is the consistency and accuracy of the individuals conducting the measurements [19]. While experienced raters often perform muscle imaging assessments in research labs and clinical settings, incorporating less experienced raters in the process is relevant and sometimes necessary. In many labs and clinical settings, expert raters may not always be available, making it essential to train raters without compromising measurement accuracy. Research has shown that variability between raters, especially those with limited experience, can be significant [24–26], showing a need for further investigation. Understanding how reliably assessors with limited experience can measure intrinsic foot muscle volume using US and MRI will provide valuable insight into their role in clinical and research settings, ensuring accuracy while expanding accessibility. Importantly, while the present study used an experienced sonographer for image acquisition to standardize image quality, our goal is to evaluate whether assessors with limited experience can reliably perform the downstream segmentation and volume calculations using a protocol designed to be teachable and reproducible.

Assessing intrinsic muscles efficiently and with less expensive technology can benefit both the provider and the patient in a clinic setting [27]. Since ultrasound provides a timely and cost-effective source of muscle imaging, understanding its quality while measuring smaller muscles of the body is significant [27]. Up to 80 percent of people experience some kind of foot pathology during their life span [28]. Common foot conditions causing pain, neuropathy, and deformities are commonly linked to atrophy within the intrinsic foot muscles of older populations [29]. Thus, assessing the validity and reliability of intrinsic foot muscle volume measurement in older populations is important because it may allow for enhancement of clinical assessment based on muscle volume measurements.

Given the role of intrinsic foot muscles in daily function and clinical pathology, the purpose of this study was to evaluate the reliability and criterion validity of ABDH muscle volume measurements derived from US and MRI images by assessors with limited experience in image-analysis. We hypothesized that US-derived muscle volume measurements would demonstrate strong linear associations and acceptable agreement with MRI-derived measurements and would be statistically equivalent within a defined margin. This study extends prior work by focusing specifically on whole-muscle ABDH volume and the use of assessors with limited experience in muscle segmentation and volume calculation.

## Methods

Twenty-two participants were recruited for measurement of right and left ABDH muscle volumes measured via US and MRI (females = 12, males = 10) (Age 54.6 ± 6.63; Ht: 174.0 ± 9.3 cm; Wt: 84.5 ± 15.4 kg), a potential total of 44 feet. Of these, 7 feet were excluded due to protocol deviations or imaging artifacts. This resulted in a final dataset of 37 feet from 22 participants (17 right and 20 left feet). One right foot was missing the primary US measurement, resulting in 36 complete MRI-US observations. Exclusions included one participant who was removed from the MRI portion of the study due to a metal device in their neck which was not disclosed prior to the US session. Additional feet were excluded from analysis due to excessive movement during scanning that made distinguishing muscle borders in those images unfeasible or because of a marking error that occurred in a few participants resulting in incongruency in the number of slices (imaging frames) between MRI and US.

The inclusion criteria included adults 45 years of age or older with no lower extremity injury within the past month or leg/foot surgery within the past three months, except for cases of plantar fasciitis diagnosed by a US board-certified physician or podiatrist. Additionally, individuals who were pregnant, equipped with a pacemaker or implantable cardioverter-defibrillator, or presented any other contraindications for MRI were excluded from the study.

The study protocol was approved by the Brigham Young University Institutional Review Board (IRB2022−332) and written informed consent was obtained from all participants before participation in accordance with ethical guidelines. Study participants were recruited from 02 September 2022 to 01 September 2023. Demographic information was recorded for each participant. The entire imaging process took place during a single day, divided into two sessions. Both sessions were completed within one hour of each other.

### Preparation for imaging sessions

The anatomical locations for measurements with both US and MRI were established by creating a single mark at the most prominent part of the navicular tuberosity. Subsequently, a stencil with slits every 0.5 cm was positioned directly below this marking, towards the plantar surface of the foot. Additional marks were then placed along this stencil every 0.5 cm along the medial border of the foot. These markings were used in US imaging and provided guidance for the placement of a fitted gel capsule strip used in MRI. The stencil was used to minimize potential errors in markings on each foot of every participant.

### US protocol

All US imaging was acquired by a single sonographer with >3 years of experience in intrinsic foot muscle imaging to standardize acquisition quality across participants; however, the acquisition protocol was intentionally designed to be transferable to newly trained ultrasound technicians through explicit landmarking, fixed slice intervals, and standardized positioning and machine settings. All images were captured using an ML6‐15‐D matrix linear transducer probe (LOGIQ FORTIS; GE Healthcare, Chicago, IL) during the US imaging sessions. Ultrasound imaging was standardized with a scanning depth of 3 cm and a frequency of 8 MHz, with gain adjustments made to optimize image quality. During imaging sessions, each participant sat in a semi-upright position with a pillow positioned beneath the thigh of the leg being imaged. The hip of the scanned leg was abducted, and the foot was placed off the table at 90°. This positioning ensured consistency in capturing images of the medial/plantar regions of the foot for all participants.

The US probe in short-axis orientation was slid across the muscle from the proximal to the distal attachment, recording a cine-loop to show the alterations in CSA and shape of the ABDH muscle while at rest. This cine-loop served as a reference during analysis, ensuring accurate visualization of the CSA changes and aided in segmenting muscle borders. Multiple cross-sectional images of the ABDH were taken at 0.5 cm intervals along the entire length of the muscle. Between 15–25 still images, based on the length of the participants’ feet, were independently recorded for subsequent analysis. These images were captured separately after the proximal to distal video recording.

### MRI protocol

Each participant’s feet underwent imaging using an 8-channel dedicated foot/ankle coil (Knee-Foot Array, ScanMed, Omaha, Nebraska) within a 3 Tesla MRI scanner (VIDA, Siemens, Erlangen, Germany) with a Tim Trio Connector (Siemens, Erlangen, Germany). A 3D spoiled gradient echo sequence captured images of intrinsic foot muscles, with the through-plane direction set perpendicular to the foot’s long axis. Water band excitation was used to eliminate fat signal, enhancing muscle border visibility. Imaging parameters included a 0.5 mm slice thickness, contiguous slices, and a 480x296x220 matrix size. Resolution was 0.52x0.50x0.51 mm, with a 240x154x110 mm field of view. Additional parameters: flip angle 9°, no acceleration, 270 Hz/pixel bandwidth, and a total acquisition time of 10 min 11 sec per foot (TE/TR: 10.0/4.92 ms).

Participants completed an MRI safety screening before entering the magnet room. After approval, 125 mcg vitamin D3 gel capsules were affixed to the medial foot using adhesive strips at the same locations as the US session for consistency. These capsules served as visible markers in MRI (Fig 1). Imaging took 30 minutes per participant for both feet, capturing 480 coronal images per foot. The number of images measured ranged from 15–25, aligning with the US images. Muscle volume was calculated using OsiriX semi-auto volume segmenter software (OX) (OsiriX MD v.10.0.1, Pixmeo, Geneva, Switzerland) and the truncated cone formula.

**Fig 1:**
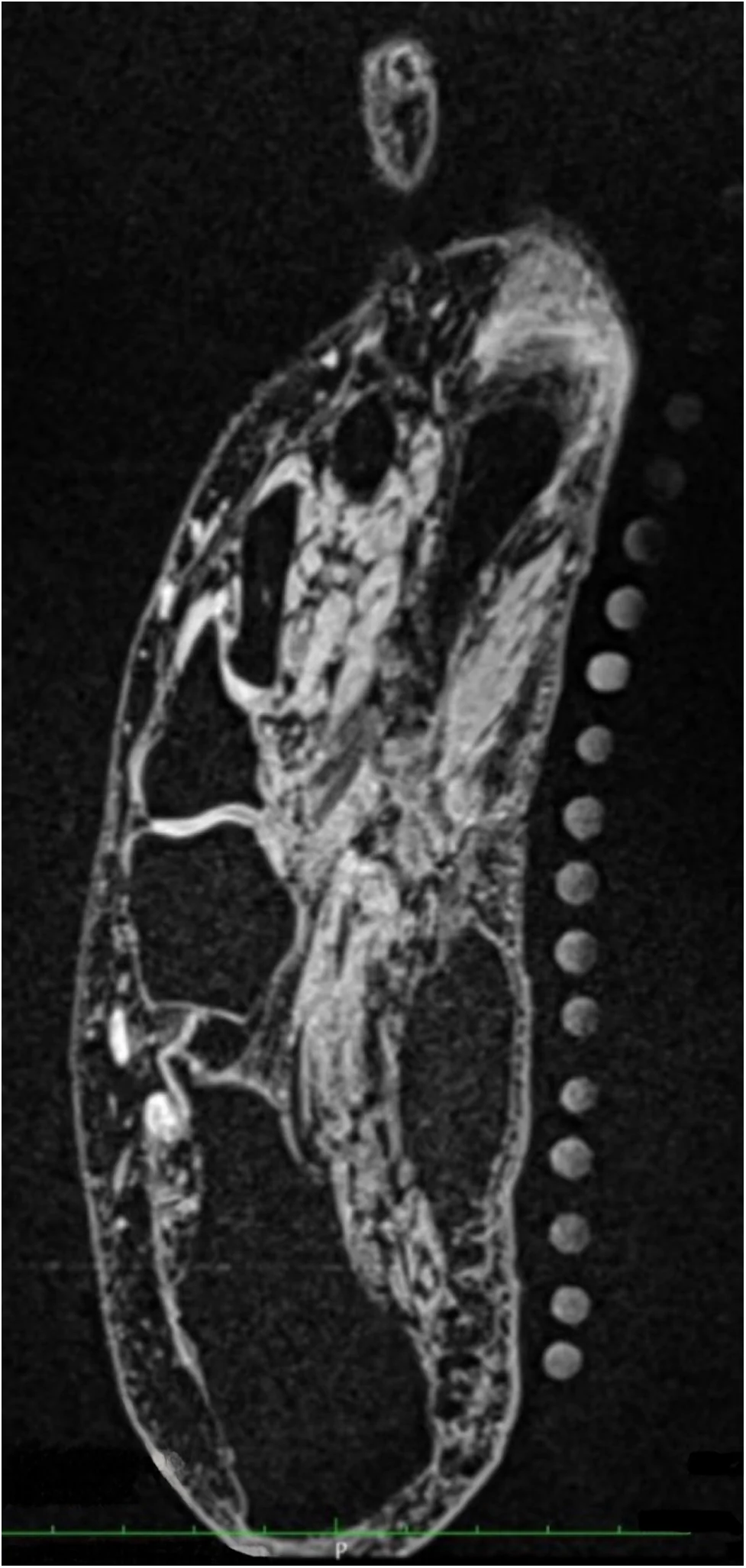
MRI with Gel Capsule Markers.

### Data processing

All MRI and US images were analyzed by assessors who had completed standardized training prior to analysis. Before study initiation, the assessors had fewer than 25 hours of experience performing muscle segmentation and volume calculations. The standardized training was designed to ensure consistent identification of muscle borders and measurement procedures.

MRI and US images were measured at intervals of 0.5 cm from the origin to the insertion of the ABDH. The individual US cross-sectional slices of the ABDH were segmented on the inner side of the muscle fascia border using the LOGIQ S8 US system. When needed, the recorded short-axis cine-loop was referred to help visualize fascial borders. The MRI slices were segmented on the visible muscle border using OX software. We used the truncated cone formula (TC) to estimate ABDH volume from both US and MRI derived ABDH CSA measurements. Additionally, we used OX to obtain another estimation of muscle volume from the MR images. The total number of cross-sectional images taken per foot ranged from 15 to 25 measurements depending on an individual’s muscle length.

US derived CSA measurements were taken from images captured in the coronal plane at the measurement locations marked on the foot as described previously. For MRI CSA measurements, readings were taken at two specific points: at the middle of the gel capsule (The middle is identified as the largest part of the capsule on the slice images) and at the midpoint between two gel capsules (Fig 2). Measurements were repeated twice for all three imaging groups: MRI OX, MRI truncated cone (MRI TC) and ultrasound truncated cone (US TC). All MRI measurements were done by the same author, and all US measurements were done by another author. Intraclass correlation coefficients (ICC) were subsequently computed based on these measurements from both MRI and US.

**Fig 2:**
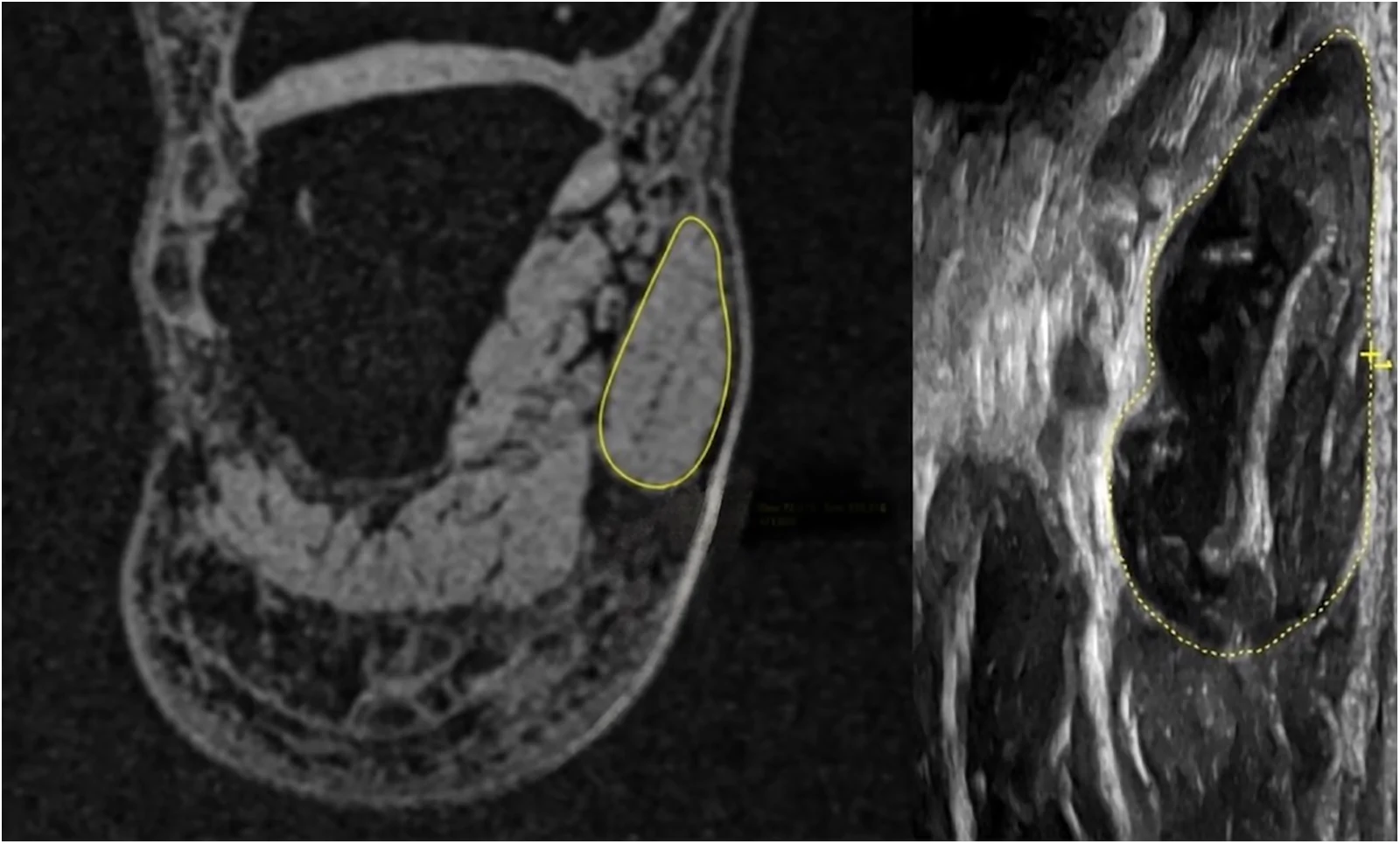
Comparison of MRI and US Derived ABDH Muscle Segmentation.

Whole muscle volume for the MRI TC and US TC methods was calculated by summing the estimated volume of each muscle segment that was created between each image slice. Muscle segment volume was estimated using the truncated cone formula:


$$\begin{array}{c} V\text{\textasciitilde{}}=\text{\textasciitilde{}}\left(h/3\right)\text{\textasciitilde{}}{(A}_{1}\text{\textasciitilde{}}+\text{\textasciitilde{}}A_{2}\text{\textasciitilde{}}+\text{\textasciitilde{}}\surd \left(A_{1}A_{2})\right) \end{array}$$

This formula has been employed in previous studies to measure the volume of various muscles in the body. In this formula, A_1_ represents CSA from the proximal image slice, A_2_ represents the CSA from the distal image slice, and h represents the slice interval or distance between the 2 slices.

OsiriX volume was estimated using the Cavalieri principle. Although MRI images were acquired as a contiguous stack with a 0.5-mm slice thickness, only selected slices corresponding to the ultrasound measurement locations were manually segmented for volume calculation. Gel capsules positioned at 1-cm intervals served as MRI-visible landmarks. Cross-sectional area measurements were obtained at the center of each gel capsule and at the midpoint between adjacent capsules, resulting in measurements at 0.5-cm intervals corresponding to the ultrasound measurement locations. Whole-muscle volume was then estimated by multiplying each segmented cross-sectional area by the 0.5-cm interval between analyzed slices and summing these values across the length of the ABDH.

### Statistical analysis

All statistical analyses were performed using SPSS version 27.0 statistical software (IBM Corporation, Armonk, NY) and R version 4.5.2 (R Foundation for Statistical Computing, Vienna, Austria). An alpha of 0.05 was employed to determine statistical significance. To account for the non-independence of bilateral feet, reliability, correlation, Bland-Altman, and method-comparison analyses were conducted separately for right and left feet.

Intraclass correlation coefficients (ICCs) with random participants and fixed raters (model 3,1) were calculated with 95% confidence intervals to assess the repeatability of muscle volume measurements for both MRI methods (MRI OX and MRI TC) and the US method (US TC). Measurement reliability was further assessed by using the standard error of measurement (SEm) and the minimal detectable change (MDC) at the 95% confidence level. SEm and MDC statistics were calculated using the following equations:


$$\begin{array}{c} SEm\text{\textasciitilde{}}=\text{\textasciitilde{}}SD\times \surd \left(1-ICC\right) \end{array}$$

$$\begin{array}{c} MDC\text{\textasciitilde{}}=\text{\textasciitilde{}}SEm\times 1.96\times \surd 2 \end{array}$$

Criterion validity of ultrasound-derived muscle volume measurements relative to MRI was evaluated using multiple complementary statistical approaches. Pearson product-moment correlation coefficients were calculated to quantify the strength of the linear association between ultrasound- and MRI-derived muscle volume measurements. Agreement between methods was evaluated using Bland-Altman analyses, which were used to identify systematic bias, estimate the limits of agreement, and visualize measurement variability between MRI OX, MRI TC, and US TC (Fig 3). Percentage differences based on the limits of agreement were calculated to quantify numerical differences between imaging methods. Differences among measurement methods were evaluated separately by side using repeated-measures analysis of variance followed by Holm-adjusted pairwise comparisons.

**Fig 3:**
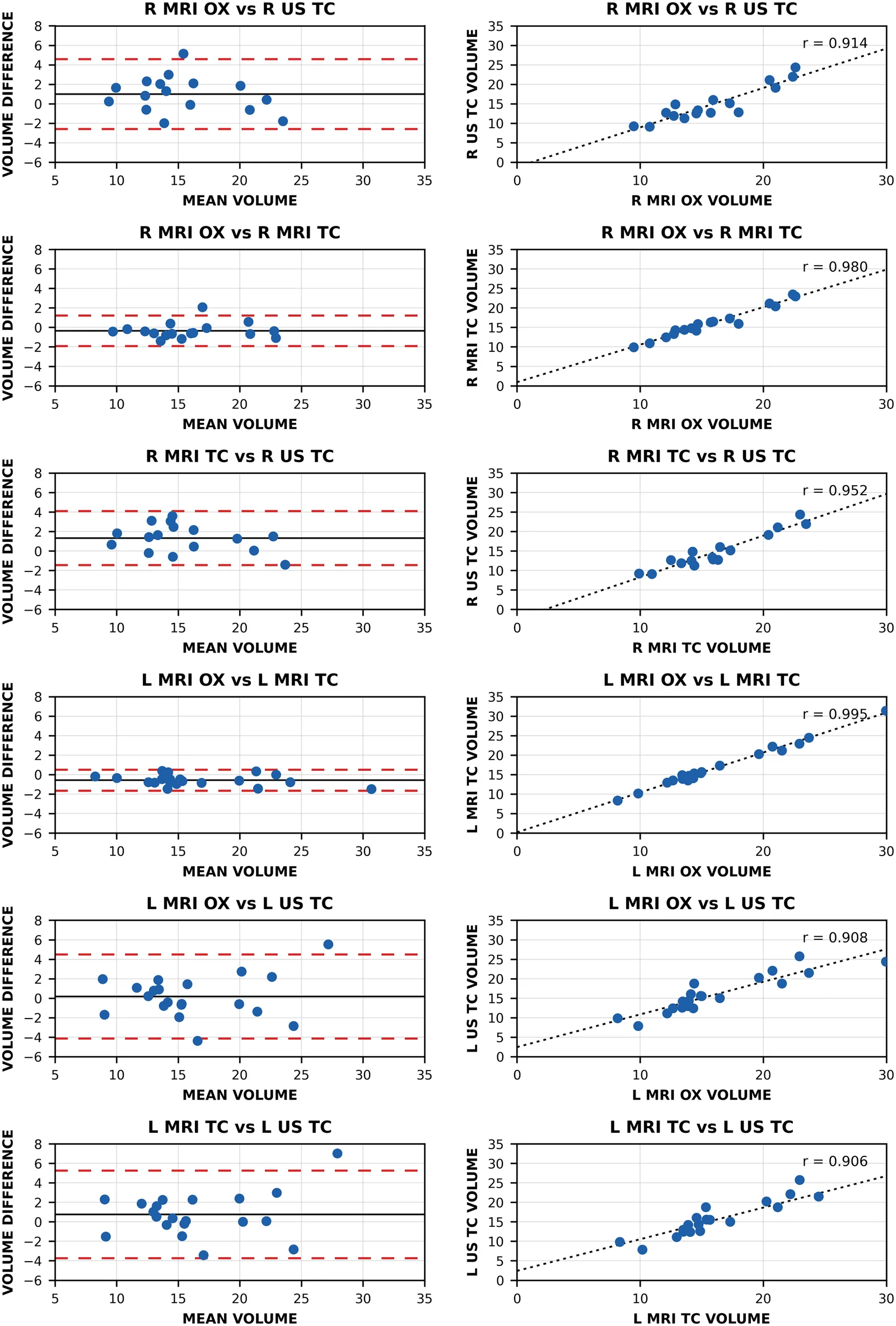
Bland-Altman and correlation plots.

Equivalence was evaluated using log-transformed generalized estimating equation (GEE) models with participant-level clustering, an exchangeable working correlation structure, and robust standard errors to account for within-participant correlation between bilateral feet. Two one-sided tests (TOST) were used to evaluate equivalence using a relative margin of ±10%. Because no established clinically meaningful equivalence margin exists for ABDH muscle volume, the ± 10% margin was selected based on expected measurement error and previous muscle volume measurement literature [16,30]. Equivalence was concluded when the 90% confidence interval for the method ratio fell entirely within the corresponding equivalence bounds of 0.90 to 1.10.

### Artificial intelligence tool usage

OpenAI ChatGPT (GPT-5.6 Sol; OpenAI, San Francisco, CA, USA) was used to generate R code used in statistical analysis, and to assist with language and grammar editing in the text of the manuscript. AI generated code used was executed in R by the authors and resulting outputs were reviewed against the study data. Any AI text suggestions used were reviewed and edited by the authors to ensure accuracy. The hypotheses, interpretations, and conclusions presented in this manuscript were conceived by the authors and represent their work.

## Results

Side-specific repeatability was high across measurement methods. Right-foot ICC_(3,1)_ values were 0.962 for MRI-OX, 0.946 for MRI-TC, and 0.997 for US-TC, while left-foot values were 0.993, 0.991, and 0.932, respectively (Table 1). SEm values ranged from 0.251 to 0.709 cm³, and MDC values ranged from 0.697 to 1.965 cm³. Relative MDC values ranged from 4.48% to 12.95% across methods and sides (Table 1). MRI repeatability estimates were based on 9–10 paired remeasurements per side, whereas US estimates were based on five right-foot and four left-foot remeasurements and should therefore be interpreted cautiously.

**Table 1:** Side-specific repeatability of ABDH muscle volume measurements.

Side	Measurement Technique & Modality	Paired remeasures, n	ICC (3,1)	SEm (cm^3^)	Relative MDC (%)	Absolute MDC (cm^3^)
Right	MRI – OsiriX	10	0.962	0.609	11.23	1.689
Right	MRI – Truncated Cone	9	0.946	0.709	12.95	1.965
Right	US – Truncated Cone	5	0.997	0.251	4.48	0.697
Left	MRI – OsiriX	10	0.993	0.462	7.94	1.280
Left	MRI – Truncated Cone	9	0.991	0.565	9.22	1.567
Left	US – Truncated Cone	4	0.932	0.347	6.82	0.963

Very strong linear associations were observed among measurement methods, with Pearson correlation coefficients ranging from 0.906 to 0.995 for corresponding side-specific comparisons (Table 2). Mean ABDH muscle volumes ranged from 14.89 to 16.83 cm³ across methods and sides (Table 3). Right-foot MRI-TC volumes were significantly greater than US-TC volumes (Holm-adjusted *p* = 0.006), whereas the other right-foot pairwise comparisons were not significant (Table 4). Left-foot MRI-TC volumes were significantly greater than MRI-OX volumes (Holm-adjusted *p* < 0.001), while the other left-foot pairwise comparisons were not significant (Table 4). Paired right-versus-left comparisons showed no significant side differences for MRI-OX (*p* = 0.387) or MRI-TC (*p* = 0.654); however, US-TC volumes were lower in the right foot than in the left foot (mean difference = −0.74 cm³, *p* = 0.047).

**Table 2:** Pearson correlations between ABDH muscle volume measurement methods.

Side	Comparison	n	r	95% CI	p
Right	MRI OX vs MRI TC	17	0.980	0.943–0.993	<0.001
Right	MRI OX vs US TC	16	0.914	0.766–0.970	<0.001
Right	MRI TC vs US TC	16	0.952	0.863–0.983	<0.001
Left	MRI OX vs MRI TC	20	0.995	0.987–0.998	<0.001
Left	MRI OX vs US TC	20	0.908	0.778–0.963	<0.001
Left	MRI TC vs US TC	20	0.906	0.773–0.962	<0.001

**Table 3:** Mean muscle volume (cm^3^).

Measurement Technique & Modality	Right Foot	Left Foot
MRI – OsiriX	15.78 ± 3.98 (n = 17)	16.25 ± 5.26 (n = 20)
MRI – Truncated Cone	16.13 ± 3.91 (n = 17)	16.83 ± 5.41 (n = 20)
US – Truncated Cone	14.89 ± 4.52 (n = 16)	16.08 ± 4.86 (n = 20)

**Table 4:** Volume comparisons between different measurement techniques.

Measurement Technique & Modality	Mean difference (cm³)	Holm-adjusted p
**Right Foot (n = 16)**		
MRI OsiriX to Ultrasound	0.99	0.094
MRI Truncated Cone to Ultrasound	1.32	0.006
MRI OsiriX to MRI Truncated Cone	−0.33	0.129
**Left Foot (n = 20)**		
MRI OsiriX to Ultrasound	0.18	0.725
MRI Truncated Cone to Ultrasound	0.76	0.315
MRI OsiriX to MRI Truncated Cone	−0.58	<0.001

Bland-Altman analyses demonstrated tighter absolute agreement between the two MRI approaches than between MRI and US (Table 5). MRI-OX versus MRI-TC biases were −0.35 cm³ for the right foot and −0.58 cm³ for the left foot, with limits-of-agreement half-widths corresponding to 9.84% and 6.52% of mean volume, respectively. MRI–US comparisons showed wider limits of agreement, with half-widths ranging from 17.86% to 27.35% of mean volume.

**Table 5:** Bland-Altman agreement between volume estimation methods.

Measurement Technique & Modality	n	Bias (cm³)	Lower LoA	Upper LoA	LoA half-width (% mean)
**Right Foot**					
MRI OsiriX to Ultrasound	16	0.99	−2.60	4.58	23.34%
MRI Truncated Cone to Ultrasound	16	1.32	−1.46	4.10	17.86%
MRI OsiriX to MRI Truncated Cone	17	−0.35	−1.92	1.22	9.84%
**Left Foot**					
MRI OsiriX to Ultrasound	20	0.18	−4.14	4.50	26.72%
MRI Truncated Cone to Ultrasound	20	0.76	−3.75	5.26	27.35%
MRI OsiriX to MRI Truncated Cone	20	−0.58	−1.66	0.50	6.52%

In the participant-clustered equivalence analysis, all three method comparisons met the defined ±10% equivalence criterion (Table 6). The MRI-TC/MRI-OX ratio was 1.031 (90% CI, 1.021–1.041; TOST *p* < 0.001), the US-TC/MRI-OX ratio was 0.964 (90% CI, 0.932–0.996; *p* < 0.001), and the US-TC/MRI-TC ratio was 0.935 (90% CI, 0.905–0.966; *p* = 0.027).

**Table 6:** Equivalence analysis between ABDH muscle volume measurement methods.

Method Comparison	Method Ratio	90% CI	Equivalence Bounds	TOST *p*-value	Equivalent
MRI-TC / MRI-OX	1.031	1.021–1.041	0.90–1.10	<0.001	Yes
US-TC / MRI-OX	0.964	0.932–0.996	0.90–1.10	<0.001	Yes
US-TC / MRI-TC	0.935	0.905–0.966	0.90–1.10	0.027	Yes

## Discussion

The objective of this study was to evaluate the reliability and criterion validity of ABDH muscle volume measurements derived from US and MRI images by assessors with limited experience in image-analysis. Measurements demonstrated high repeatability and strong linear associations across methods, and all method comparisons met the ± 10% equivalence criteria after accounting for bilateral clustering. However, Bland–Altman analyses demonstrated tighter absolute agreement between the two MRI methods than between MRI and US, and statistically significant systematic differences were present for some method comparisons. These findings support criterion validity within the specified equivalence margin but do not indicate that the methods produce identical or fully interchangeable individual measurements. These findings are consistent with prior work demonstrating strong associations between MRI- and ultrasound-derived intrinsic and extrinsic foot muscle CSA measurements [15] and with studies demonstrating that assessor experience can influence measurement reliability [24–26].

Previous research has consistently shown a positive correlation between experience in image analysis and the accuracy of muscle size measurements, with experienced raters typically achieving more precise results. Fortin et al. [25] found that experienced raters achieved an ICC between 0.94 and 0.99, compared to 0.84 and 0.99 for novice raters when using MRI to take CSA measurements of cervical spine extensor muscles, indicating a difference in measurement reliability. Johnson et al. [26] demonstrated that the use of cine loops in ultrasound imaging improved measurement reliability when examining muscle size changes in the lower leg and foot, particularly when assessed by novice raters. In novice raters, the use of cine loops in the measurement of various foot muscles decreased the intraoperator standard error by 45–73% and limits of agreement by 24–51%, resulting in more comparable measurements between novice and experienced raters. Similarly, Carr et al. [24] reported a statistically significant difference between images analyzed by experienced versus novice raters for vastus lateralis CSA (p = 0.005). These referenced studies assessed CSA, while the current study assessed muscle volume.

Additional work examining imaging modality validity in muscle volume has demonstrated that ultrasound and MRI can be highly reliable when standardized protocols are used. Swanson et al. [31] reported excellent repeatability and reliability for plantar fascia thickness and flexor digitorum brevis muscle volume measurements, with ICC values ranging from 0.989–0.997 across imaging techniques and strong correlations between ultrasound and MRI measurements (r = 0.94–0.99). Unlike this prior work, the present study specifically evaluated ABDH muscle volume measurements performed by assessors with limited segmentation experience.

These findings are relevant for labs and clinical settings with teams of varying experience levels. Inexperienced raters can introduce variability and reduce the reliability of measurements, impacting the validity of study results and clinical measurements, potentially affecting clinical decisions and patient outcomes. To mitigate these effects, it is essential to consider rater experience in study design and implement adequate training and standardization protocols to enhance measurement accuracy across all experience levels.

Ultrasound is increasing in its clinical use within orthopedic, musculoskeletal and rehabilitation settings [32] as a point of care modality to assess injury, document improvement and for injection guidance. This increase in ultrasound use is due to portability and accessibility of equipment, cost savings and time efficiency. A major benefit of ultrasound assessment is dynamic real time imaging and testing, along with the use of cine loops (short, recorded videos). When measuring muscles, cine loops are commonly used to capture a sequence of ultrasound images showing the muscle transitioning from a relaxed state to a contracted position and then returning to relaxation [26]. In this study, a resting proximal-to-distal cine loop was available as a reference during segmentation; however, a contract-relax cine-loop protocol was not used. Use of a contract-relax cine loop may further improve border identification and measurement precision.

Measurement of muscle size and volume is a process that can take longer periods of time due to the need of manually measuring many CSAs per muscle, particularly for those longer in length. In emergency and clinical settings, it is important to not only make sure those measurements are accurate and precise but also calculated very quickly to ensure the health and quality care of the patient. Machine learning and artificial intelligence are continually being investigated as a tool used to decrease time spent gathering these measurements.

### Limitations

One limitation of this study is that it focused on an older population. Age-related changes in muscle composition and volume associated with sarcopenia, particularly muscle atrophy, fibrosis and fatty infiltration, could potentially increase the difficulty of measuring CSAs. These factors increase difficulty of identifying muscle borders and potentially alter the expected shape of the muscle being segmented. However, the older population is clinically relevant because this population experiences higher rates of pathology compared to younger populations. Despite these challenges, the measurements demonstrated high repeatability and met the equivalence criterion, although MRI-US comparisons demonstrated wider limits of agreement than MRI-MRI comparisons, indicating greater individual-level variability.

Another methodological limitation involves our use of static US images for CSA measurement with referral back to a single cine loop of the muscle at rest moving from proximal to distal across the muscle belly. This is a relevant issue as previous studies have shown increased reliability while using a contract-relax cine loop [26]. Less experienced raters using static images to segment muscle size may have greater difficulty in identifying muscle borders. The use of contract-relax cine loops allows for dynamic imaging and easier identification of muscle borders, which may reduce measurement error. Despite this limitation, our study still resulted in high reliability metrics. When contract-relax cine loops are not feasible in clinical or research settings, static images with a resting cine loop available for reference may still provide repeatable estimates of muscle size.

## Conclusion

ABDH muscle volume measurements derived from US and MRI images by assessors with limited prior image-analysis experience demonstrated high repeatability, strong linear associations, and statistical equivalence within the ± 10% margin. However, MRI-US measurement comparisons showed wider limits of agreement than comparisons between the two MRI methods, indicating greater variability at the individual-measurement level. These findings support US as a potential practical alternative for ABDH volume assessment when MRI is impractical while recognizing method-specific measurement variability. Future studies should examine whether additional training, contract-relax cine loops, or automated segmentation methods can further improve measurement precision.
